# Editorial: Greenhouse gases mitigation strategies in grazing ruminants

**DOI:** 10.3389/fvets.2024.1360276

**Published:** 2024-01-16

**Authors:** Paulo de Mello Tavares Lima, Tiago do Prado Paim, Tim McAllister

**Affiliations:** ^1^Department of Animal Science, University of Wyoming, Laramie, WY, United States; ^2^Goiano Federal Institute of Education, Science and Technology (IF Goiano) Campus Rio Verde, Rio Verde, GO, Brazil; ^3^Agriculture and Agri-Food Canada, Lethbridge, AB, Canada

**Keywords:** cattle, livestock, methane, sheep, sustainability

Cultivated and natural grasslands from both tropical and temperate countries play a crucial role for the subsistence of rural communities all over the world by supporting ruminant livestock in developed and developing countries (1). Higher demand for animal food products such as milk and meat by 2050 will drive intensification, which also includes intensification of grazing system as a key component of ruminant production and a challenging sector to mitigate greenhouse gas (GHG) emissions (2, 3). Methane (CH_4_) is one of the most important environmental concerns associated with ruminant production, with enteric fermentation from ruminant livestock being responsible for 30% of anthropogenic emissions (4). Inclusion of cereal grains or oils in ruminant diets can reduce the intensity of emissions (GHG/unit of animal product) as compared to grazing ruminants, by increasing gain and reducing the amount of CH_4_ per unit of feed digested (5). However, feeding grains in extensive grazing systems can be difficult or even impossible (4). Reducing GHG emissions from grazing ruminants has proven to be a challenge and is a priority of the industry. This Research Topic generated five manuscripts that describe integrated animal—plant management practices, as well as knowledge on the rumen microbiome that can be used to mitigate GHG emissions from grazing ruminants.

Intensification of ruminant grazing systems include approaches related to both pasture management and animal genetics. Oliveira et al. undertook a comprehensive study in the Brazilian Atlantic Forest biome in factorial design (2 × 2) where two different genotypes of dairy cows were evaluated (Holstein and crossbred Holstein × Jersey) under two different grazing systems (Continuous with low stocking rate × rotational with high stocking rate). These authors measured GHG emissions in these systems (CH_4_ and nitrous oxide) and observed that regardless of breed and pasture management, soil carbon sequestration was not enough to neutralize emissions from any of the systems. However, they estimated the potential of planting trees in these systems to neutralize emissions, highlighting the potential of silvo-pastoralism to enhance carbon sequestration and the sustainability of grazing systems. Planting trees in grazing systems was able to significantly reduce overall GHG emissions in all treatments.

Utilization of locally available agricultural co-products is often pointed out as a strategy of promoting sustainability in animal production systems (6). Such an approach frequently lowers feed costs and can indirectly and directly reduce GHG emissions depending on the co-product. Budel et al. evaluated the use cakes originated after oil extraction from the Amazon fruits cupuassu (*Theobroma grandiflorum*) and tucuma (*Astrocaryum vulgare* Mart.), used in both food and cosmetic industry (7, 8). These authors explored the effect of these cakes in lamb diets using a 40:60 forage:concentrate ratio. The co-products did not change GHG emissions, digestibility, blood metabolites and growth performance. Cupuassu was similar and tucuma was superior to the control diets when analyzing CH_4_ production per unit of body weight gain. Despite the lack of studies on these co-products, their results showed that they should be suitable for utilization in grazing systems, especially during periods of forage scarcity.

Pinnell et al. used 16S rRNA sequencing to identify discriminant taxa in different rumen microenvironments. The authors highlighted that rumen microorganisms associated with CH_4_ production were more abundant in the microbial community of the fluid fraction of rumen content as compared to the microbial communities associated with the rumen mucosa or those associated with fiber particles in rumen. Even though the findings were not completely novel, the work did emphasize that the association between enteric CH_4_ emissions and the structure of ruminal microbial community can be greatly influenced by sample collection.

Liu et al. generated a review addressing gastrointestinal microbes-related factors that affect productive traits in dairy cows. Even though this manuscript has a clearer focus on dairy cows, which often are raised in confinement (9, 10), some of the aspects considered in the review are clearly applicable to grazing ruminants, especially those components that focuses on CH_4_ emissions. The manuscript describes that typically, cows with lower feed conversion rates have higher ruminal microbial diversity, with methanogens of the genus *Methanobrevibacter* predominating. The paper also emphasized the negative relationship between CH_4_ production and productivity. On that same study, authors also brought attention to the fact that feeding ruminants high forage diets, which is the case with grazing, provides substrates that promotes the growth of a variety of beneficial microorganisms in the rumen, but at the disadvantage of also increasing the activity of those microbiota involved in CH_4_ production. For this reason, the authors suggest the utilization of feed additives such as the 3-nitrooxypropanol (3-NOP) (11) to reduce CH_4_ emissions, although such additives cannot be easily administered to grazing ruminants.

Finally, Smith et al. presented a review focusing on general aspects related to enteric CH_4_ research from pasture-fed ruminants, detailing numbers related to the representability of this gas on global GHG balances, describing physiological processes associated with CH_4_ production, and also providing an embracing overview of mitigation practices. The authors called particular attention to strategies that optimize animal growth, such as providing forages of improved nutritional quality, which may lead to reduced intensity and lifetime GHG emissions. Special attention was also given to the genetic selection of low-emitting animals as a promising strategy to abate GHG emissions. This review also addressed the main characteristics of technologies available for CH_4_ quantification, which can be of particular interest for researchers that wish to attempt to measure GHG in grazing ruminant production systems.

Overall, this Research Topic brought an updated overview about management practices and knowledge on GHG emissions from grazing ruminants, providing significant contribution to this research area.

## Author contributions

PMTL: Writing—original draft, Writing—review & editing. TPP: Writing—review & editing, Writing—original draft. TM: Writing—review & editing, Writing—original draft.
